# Organic Electrochemical Transistors/SERS-Active Hybrid Biosensors Featuring Gold Nanoparticles Immobilized on Thiol-Functionalized PEDOT Films

**DOI:** 10.3389/fchem.2019.00281

**Published:** 2019-04-26

**Authors:** Jia-An Chou, Chieh-Lin Chung, Po-Cheng Ho, Chun-Hao Luo, Yu-Han Tsai, Chung-Kuan Wu, Chiung-Wen Kuo, Yu-Sheng Hsiao, Hsiao-hua Yu, Peilin Chen

**Affiliations:** ^1^Department of Materials Engineering, Ming Chi University of Technology, New Taipei City, Taiwan; ^2^Institute of Chemistry, Academia Sinica, Taipei, Taiwan; ^3^Division of Nephrology, Department of Internal Medicine, Shin-Kong Wu Ho-Su Memorial Hospital, Taipei, Taiwan; ^4^Research Center for Applied Sciences, Academia Sinica, Taipei, Taiwan

**Keywords:** gold nanoparticles, poly(3, 4-ethylenedioxythiophene), bioelectronic interfaces, organic electrochemical transistors, surface-enhanced Raman scattering

## Abstract

In this study we immobilized gold nanoparticles (AuNPs) onto thiol-functionalized poly(3,4-ethylenedioxythiophene) (PEDOT) films as bioelectronic interfaces (BEIs) to be integrated into organic electrochemical transistors (OECTs) for effective detection of dopamine (DA) and also as surface-enhanced Raman scattering (SERS)—active substrates for the selective detection of *p*-cresol (PC) in the presence of multiple interferers. This novel PEDOT-based BEI device platform combined (i) an underlying layer of polystyrenesulfonate-doped PEDOT (PEDOT:PSS), which greatly enhanced the transconductance and sensitivity of OECTs for electrochemical sensing of DA in the presence of other ascorbic acid and uric acid metabolites, as well as amperometric response toward DA with a detection limit (S/N = 3) of 37 nM in the linear range from 50 nM to 100 μM; with (ii) a top interfacial layer of AuNP-immobilized three-dimensional (3D) thiol-functionalized PEDOT, which not only improved the performance of OECTs for detecting DA, due to the signal amplification effect of the AuNPs with high catalytic activity, but also enabled downstream analysis (SERS detection) of PC on the same chip. We demonstrate that PEDOT-based 3D OECT devices decorated with a high-density of AuNPs can display new versatility for the design of next-generation biosensors for point-of-care diagnostics.

## Introduction

Dopamine (DA), a neurotransmitter, plays important physiological roles in movement, motivation, memory, and other functions. Dysfunction of dopaminergic neurons is associated with, for example, Parkinson's disease, Alzheimer's disease, bipolar disorder, and restless leg syndrome (Coyle et al., 1983; Paulus and Trenkwalder, 2006; Ziemssen and Reichmann, 2007). Moreover, determining the levels of DA in biological systems can provide valuable information for the diagnosis, treatment, and prognosis of these neurological diseases. *p*-Cresol (PC), which is highly conjugated with proteins, is a product of the intestinal bacterium metabolism of tyrosine and phenylalanine and can be excreted by the kidneys. Serum PC levels are correlated with chronic kidney disease and uremic symptoms (Bammens et al., 2003, 2006). In addition, serum PC levels also correlate with the prognosis of cardiovascular diseases. Therefore, the detection of serum PC can influence disease treatment and prevention. Furthermore, PC can inhibit dopamine-β-hydroxylase (DBH), which converts DA to norepinephrine, a small-molecule neurotransmitter. The presence of PC in the central nervous system can lead to excesses of DA and its metabolites, which are possibly associated with neurological disorders. Nevertheless, the effects of DA and PC in most diseases remain unclear, especially in neurological disorders and their presentation in chronic kidney disease. Therefore, a challenge remains to develop methods for the rapid, sensitive, and selective detection of DA and PC in biological samples, thereby enabling assessments, during routine clinical diagnoses, of the complex relationships among the expression levels of secretions, the effects of uremic toxins, and the neurological disorders.

In addition to high-performance liquid chromatography (HPLC) method, several promising analytical assays exhibiting high sensitivity and selectivity have been developed for the detection of DA and PC (Yoshitake et al., 2006; Zhou et al., 2013; Ferry et al., 2014; Pandikumar et al., 2014; Mercante et al., 2015; Yusoff et al., 2015; Sajid et al., 2016). For example, the fluorescence method, the surface-enhanced Raman scattering (SERS) detection assay, passive component measurement methods (based on electrochemical electrodes), and active component measurement methods (based on field effect transistors) have been explored widely for biosensing in the presence of ascorbic acid (AA) and uric acid (UA) metabolites. In particular, poly(3,4-ethylenedioxythiophene):polystyrenesulfonate (PEDOT:PSS)—based organic electrochemical transistors (OECTs) have recently attracted attention for their high electrochemical identification capabilities, easy operation (driven at voltages below 1 V), low cost (prepared using vacuum-free fabrication processes), and stable performance (operated under aqueous conditions) (Tang et al., 2011; Gualandi et al., 2016, 2018; Wang et al., 2018a). In addition, SERS-active substrates have been investigated thoroughly for their tremendous potential to detect complex biomaterials, due to their high chemical identification capabilities, high sensitivity, low cost, ready implementation, and less time consuming operation. Nevertheless, we are unaware of any previous reports of OECT/SERS hybrid biosensors designed specifically for investigations of the correlations between DA and PC in biological samples, although such device concepts would presumably be viable alternatives to other sensing platforms.

In the last decade, nanoparticles (NPs) have been exploited widely in electrochemical sensors to enlarge their surface areas, promote catalyzed electrochemical reactions, and enhance electron transfer at their electrode–electrolyte interfaces (Kim et al., 2010; Huang et al., 2014; Ali et al., 2018). Gold nanoparticles (AuNPs) are particularly attractive nanomaterials for electrochemical biosensing because of their efficient signal amplification, good stability, and outstanding biocompatibility in electrolyte solutions. Although AuNPs can ensure SERS detection with improved signal reproducibility and lower toxicity, AuNP-based SERS-active substrates have lower enhancement factors (EFs) than do those incorporating silver nanoparticles (AgNPs) (Ou et al., 2014; Chiang et al., 2017). To reverse the gap between the SERS signals of AuNP- and AgNP-active substrates, electromagnetic hot spots can be further created through well-controlled AuNP aggregation, resulting in greater EFs from SERS-active substrates (Guerrini and Graham, 2012; Ou et al., 2014; Yang et al., 2016; Chiang et al., 2017). The efficiency of the hot spots of SERS-active substrates can be modulated through variation of the sizes of the AuNPs and the compositions of their colloidal solutions. Nevertheless, manipulation of the uniform aggregation of AuNPs over large areas remains a key challenge when creating hot spots on SERS-active substrates (Shiohara et al., 2014). In fact, three-dimensional (3D) SERS-active substrates have recently be found to create high-density hot spots under laser illumination; because they possess large specific surface areas for the immobilization of more AuNPs and the absorption of more target analytes, such systems might solve the problem of intrinsically weak Raman signals (Lee et al., 2012; Liu et al., 2014; Chiang et al., 2017).

In this present study, we prepared an integrated 3D-OECT device featuring a AuNP-immobilized bilayer PEDOT film as the active channel; we fabricated it from a high-conductivity PEDOT:PSS underlying layer and a nanostructured thiol-functionalized PEDOT upper layer presenting AuNPs on top. We have used these OECTs for the effective detection of DA and for the preparation of SERS-active substrates for the selective detection of PC in the presence of multiple metabolites. To the best of our knowledge, this paper is the first to demonstrate OECT/SERS hybrid biosensors for the efficient detection of DA (using OECTs) with the additional capability of sensing PC (using SERS spectroscopy).

## Materials and Methods

### Chemicals and Materials

Hydroxymethyl EDOT (EDOT-OH, 95%), sodium hydride (NaH, 60% dispersion in mineral oil), trityl chloride (97%), magnesium sulfate (MgSO_4_), potassium carbonate (K_2_CO_3_), triphenylphosphine (PPh_3_), Amberlite IR-120 resin, sodium methoxide (NaOMe), iron(III) *p*-toluenesulfonate hexahydrate (technical grade), imidazole (IM, 99%), dimethyl sulfoxide (DMSO), (3-glycidoxypropyl)trimethoxysilane (GOPS, ≥98%), hydrogen tetrachloroaurate(III) hydrate (HAuCl_4_), trisodium citrate dihydrate (TSC, 99%), DA, AA, UA, and PC were purchased from Sigma–Aldrich. The aqueous PEDOT:PSS solution (Clevios PH1000, PEDOT:PSS ratio = 1:2.5) was purchased from Heraeus with a solid concentration 1.0–1.3 wt%. Tetrahydrofuran (THF), ethyl acetate (EA), hexane, acetone, carbon tetrabromide (CBr_4_), potassium thioacetate (KSAc), hydrochloric acid (HCl, 37%), and nitric acid (HNO_3_, 70%) were purchased from Acros Organics. All materials and reagents were used as received without further purification. The aqueous solutions were prepared with deionized (DI) water from a Millipore Milli-Q water treatment system (18.2 MΩ cm^−1^).

### AuNPs

Briefly, the citrate-capped AuNPs were synthesized using trisodium citrate dehydrate (TSC) and a modified version of a method reported previously (Hsiao et al., 2012). To ensure that no other metal ions were present during the synthesis of the AuNPs, all glassware and magnetic stir bars were cleaned with freshly prepared aqua regia (3:1 HCl:HNO_3_, vol/vol), washed with copious amounts of DI water, and dried prior to use. The synthesis was performed as follows: (1) individual aqueous solutions of 0.5 wt % HAuCl_4_, 1 wt % TSC, and 0.1 wt % AgNO_3_ were stored at 4°C for at least 30 min; (2) the HAuCl_4_ (2 mL) and AgNO_3_ (85 μL) solutions were mixed, and then the TSC solution (1.8 mL) was added; (3) this mixed solution was further diluted with DI water to a total volume of 5 mL, and then boiling deionized (DI) water (95 mL) was added into the 250-mL two-neck flask under stirring.

### OECTs

#### Device Fabrication

**Figure 1:**
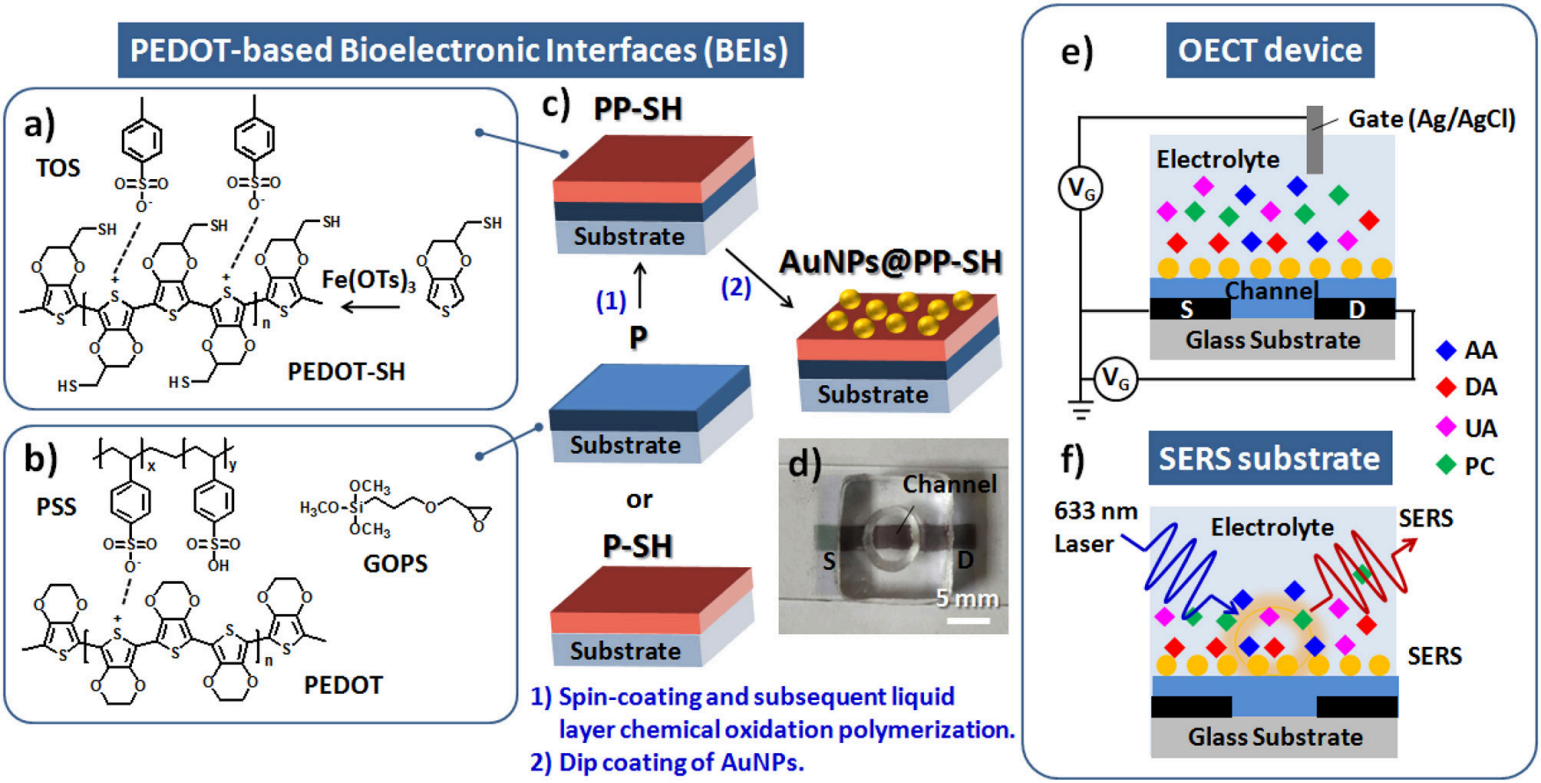
**(a)** Chemical oxidative polymerization of EDOT-SH monomers by Fe(OTs)_3_ for the fabrication of **P-SH** films. **(b)** Chemical structures in the PEDOT:PSS solution with the GOPS crosslinker for the fabrication of **P** films. **(c)** Schematic representation of four different architectures of PEDOT films (**P**, **P-SH**, **PP-SH**, **AuNPs@PP-SH**) as BEIs. **(d)** Photograph of a **AuNPs@PP-SH**–based OECT/SERS hybrid biosensor featuring a cylindrical PDMS chamber. **(e,f)** Schematic representation of a PEDOT-based OECT/SERS hybrid biosensor for OECT sensing of DA and SERS detection of PC.

The single-layer of PEDOT:PSS (with the GOPS crosslinker) and thiol-functionalized PEDOT films were individually prepared as the active layer channel materials, and denoted as **P** and **P-SH** films, respectively; the bi-layer conducting polymer film, including **P** as underlying layer and **P-SH** as top interfacial layer, was prepared as the active layer channel materials, and denoted as **PP-SH** film. The spin coating parameters were (5000 rpm, 60 s), (2000 rpm, 60 s), and (800 rpm, 180 s) for obtaining three different thicknesses (100, 200, and 400 nm, respectively) of **P** thin films; **P-SH** thin films at a thickness of approximately 90 nm were prepared by the chemical oxidative polymerization process (see Supplementary Material for detail preparation). The active layer channel of the OECTs [width (W): 1.5 mm; length (L): 5 mm] was first prepared through a sequence of coating processes (Figures 1a–c) and then by patterning **P** and **PP-SH** films using a commercial CO_2_ laser engraving system (Universal VLS2.30, Universal laser System, AZ, USA) with high-power density focusing optics (HPDFO). The device assembled from the **PP-SH**–based OECT featured a cylindrical PDMS chamber (volume: ca. 50 μL) that was used to confine the channel area of the OECT device. To prepare the OECT/SERS hybrid biosensors, the device was treated with the as-prepared aqueous AuNP solution [optical density (OD) = 0.4; 150 μL] for at least 6 h, and then washed with DI water thoroughly (five times) to remove any non-immobilized AuNPs. The resulting OECT (**AuNPs@PP-SH**) was a ready-to-use system for the detection of analytes by measuring the electrical signals and SERS spectra (see Figures 1c–f).

### Materials and Device Characterization

Details of the characterization of the materials are provided in the Supporting Information. For three-terminal OECT electrical measurements, the integrated measurement system consisted of two source meters (Keysight B1500A and Agilent B2912A) and a switching matrix (Agilent E5250A), controlled by a personal computer through customized LabVIEW software; the electrical signals of as-prepared OECT device were measured in 1× PBS (pH 7.4) buffer with Ag/AgCl wire as the gate electrode. The drain current (*I*_d_; scan rate: 12.5 mV s^−1^) was obtained by applying different source-gate voltages (*V*_g_: from −0.8 to +0.8 V) and a fixed source-drain potentials (*V*_d_: −0.4 or −0.01 V). Transconductance (*g*_m_) curves were obtained by deriving the transfer curves and the definition given in Equation (1).

(1) $$\begin{array}{r} g_{m}=\frac{\partial I_{d}}{\partial V_{g}} \end{array}$$

The electrochemical response toward DA was investigated from the (*g*_m_–*V*_g_) curves and the amperometric (*I*_d_–*t*) curve. All of the as-prepared OECT devices were stored in PBS buffer for at least 30 min prior to each testing.

For SERS measurements, Raman spectroscopy (HR800, HORIBA, Japan) was used to measure the Raman spectra of PC in the range 200–3600 cm^−1^ using a 17-mW-output helium–neon (He–Ne) laser operated at a wavelength of 633 nm. The Raman signals were collected under the conditions of a fixed 5-s exposure and accumulation four times. A constant amount of PC solution (10 μL) was placed on the as-prepared **AuNPs@P-SH** devices (area: 0.196 cm^2^) for SERS detection.

## Results and Discussion

### Characterization and Surface Morphology of PEDOT Films

**Figure 2:**
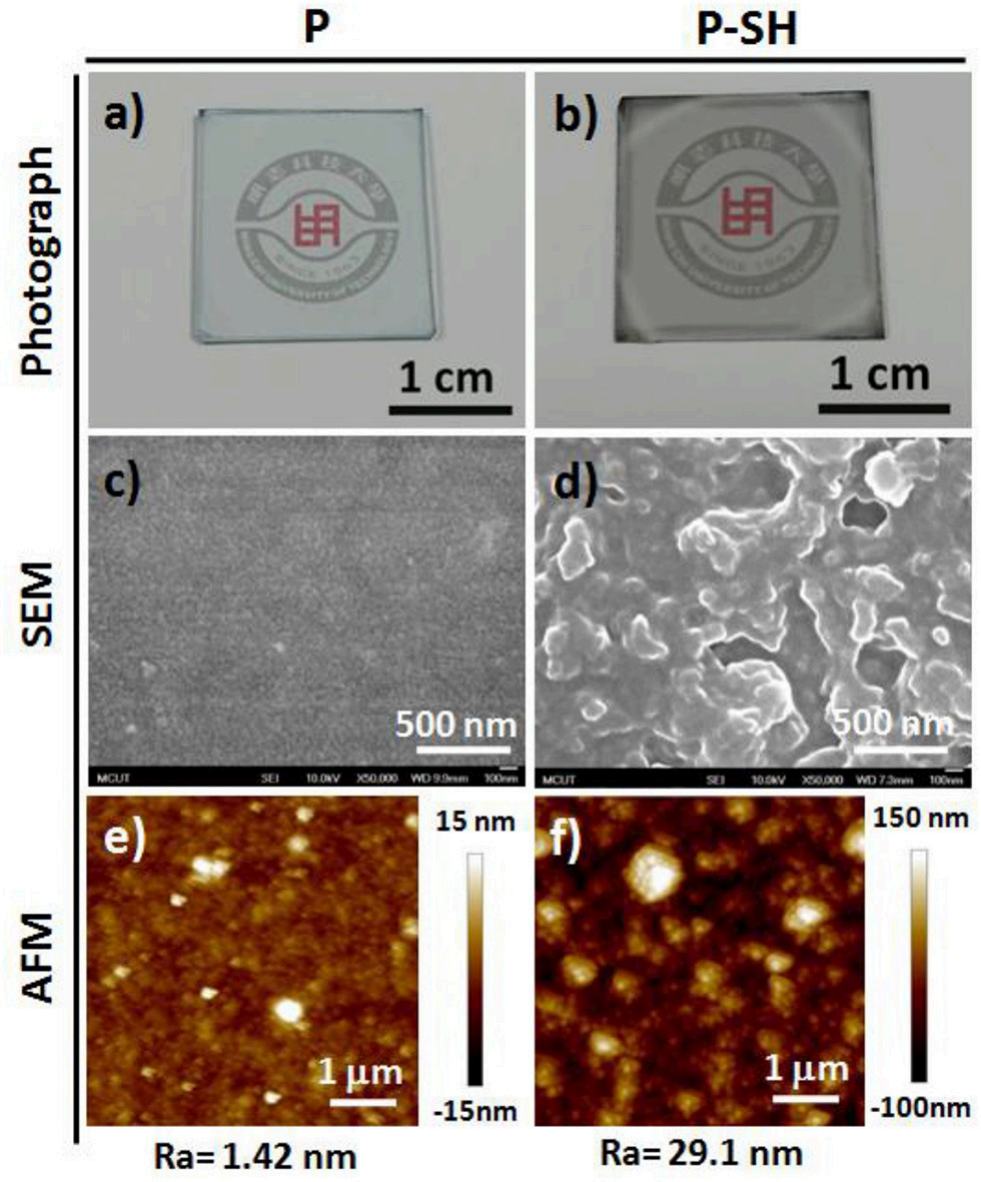
Photographs and SEM and AFM images, with mean average roughnesses (*R*_a_), of **(a,c,e) P** and **(b,d,f) P-SH** films on glass substrates.

Prior to device fabrication, we studied the optical transparency and surface morphology of the PEDOT-based bioelectronic interfaces (BEIs). Figure 2 presents photographs and SEM and AFM images of the P and P-SH films. Figures 2a,b reveal that the P and P-SH films had thicknesses of 200 and 90 nm, respectively, with both exhibiting high transparency. The oxidized polymerization process of the P-SH film, however, led to the ready formation of a 3D porous-like nanostructure, thereby giving a surface roughness (Ra = 29.1 nm) higher than that of the P films (Ra = 1.42 nm) (Figures 2c–f). Such a high surface area of the BEI could possibly improve the performance through signal enhancements in the electrochemical and SERS detections.

### Characterization of AuNPs and PEDOT Films

**Figure 3:**
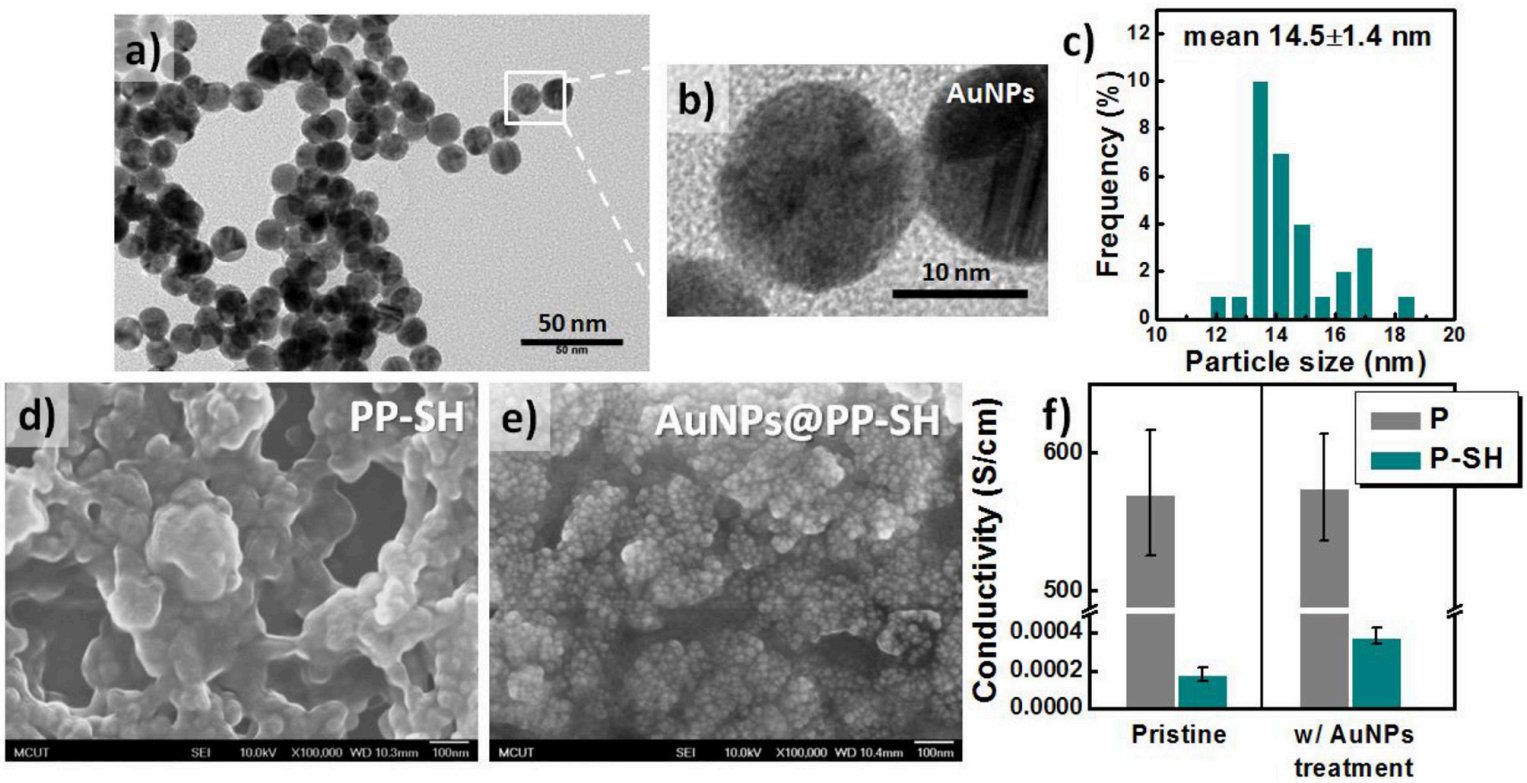
Characteristics of citrate-capped AuNPs. **(a–c)** TEM images and corresponding size distributions of citrate-capped AuNPs. **(d, e)** SEM images of **(d) PP-SH** and **(e) AuNPs@PP-SH** electrodes (with AuNPs immobilized on **PP-SH** electrodes) on glass substrates. **(f)** Electrical conductivity of **P** and **P-SH** electrodes prepared with and without AuNP treatment. The thicknesses of the **P** and **P-SH** films were ~200 and 90 nm, respectively.

To synthesize AuNPs for use in the OECT/SERS hybrid detection system, we employed a modified version of a procedure reported previously for fabricating quasi-spherical gold nanostructures (Hsiao et al., 2012). TEM analysis confirmed that the Au spheres were highly monodisperse, with a uniform size of ~14.5 ± 1.4 nm (Figures 3a–c); the UV–Vis absorption spectrum of the aqueous AuNP solution featured a strong absorption peak near 519 nm, arising from surface plasmon resonance, with a relatively narrow of full width at half maximum (FWHM), suggesting a narrow size distribution for the AuNPs (Figure S1, see Supplementary Material). After immobilizing the citrate-capped AuNPs and washing with copious amounts of DI water, SEM revealed a highly dense coating of uniformly distributed AuNPs on the P-SH film, presumably because of strong covalent bonding between the Au NPs and the thiol-functionalized P-SH films. We suspected that the surface of the AuNPs@P-SH film, with large-scale aggregation or an interconnected network of AuNPs, would provide a large electroactive surface area and more hot-spot resonance effects, thereby substantially enhancing the electrochemical and Raman signals, respectively of analytes on such chips (Figures 3d,e). Nevertheless, the electrical conductivity of the P-SH film after AuNP treatment (AuNPs@P-SH) was enhanced only slightly relative to that of the pristine P-SH film (Figure 3f). We suspect that the presence of citrate units, used for capping the AuNPs and thereby achieving a densely packed monolayer of AuNPs on the thiol-functionalized surfaces, may have led to the insignificant improvement in conductivity, due to a nonlinear *I*–*V* response, as measured using the standard four-point probe method.

### Zeta-Potentials and Electrochemical Properties of PEDOT Films

**Figure 4:**
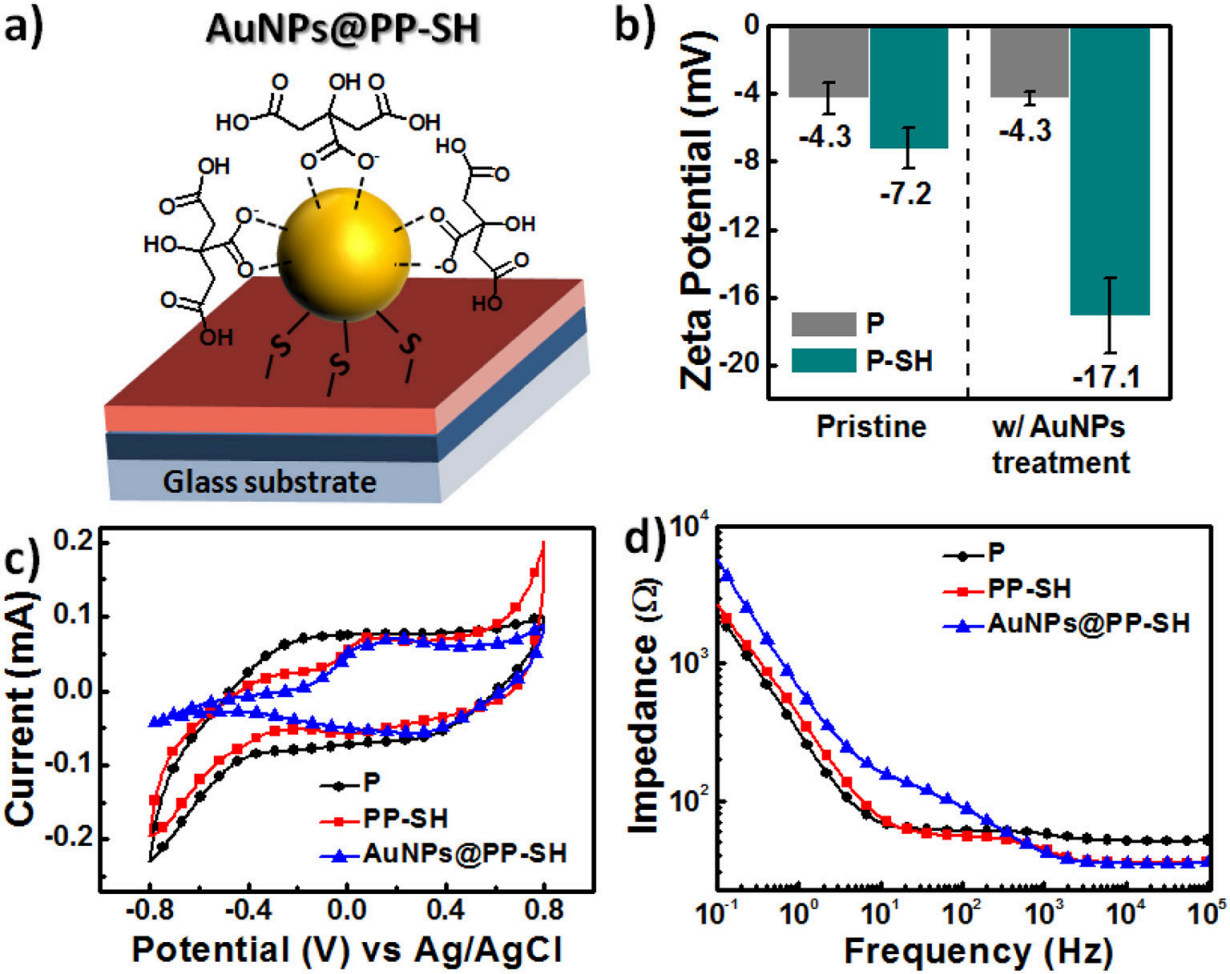
**(a)** Schematic representation of a single citrate-capped AuNP immobilized on the PP-SH film (**AuNPs@PP-SH**). **(b)** Surface charge, measured through electrokinetic analyses, on the pristine and AuNP-treated PEDOT films (**P** and **P-SH**, respectively). **(c,d)** Electrical properties of **P**, **PP-SH**, and **AuNPs@PP-SH** films in 1× PBS (pH 7.4): **(c)** CV curves (potential swept from −0.8 to 0.8 V at a scan rate of 100 mV s^−1^) and **(d)** EIS spectra (frequency range: from 10^−1^ to 10^5^ Hz).

According to the design rule of BEIs for electrochemical sensing, the presence of more negative surface charges on electrodes can be used to resolve the problem of interference from overlapping signals of DA, AA, and UA metabolites (Wang et al., 2009; Huang et al., 2014). Therefore, if the BEI is designed to favor cationic exchange—that is, to attract cationic DA and repel anionic AA and UA—it will thereby allow the selective detection of DA in the presence of AA and DA. Figure 4a presents a schematic representation of **AuNPs@PP-SH** films, which we suspected would be more suitable for preventing negatively charged AA and UA from reaching the surface, due to the presence of a surface of negatively charged citrate ions on the AuNPs. The pristine **P** and **P-SH** films both exhibited similar zeta potentials, ~-4.3 and −7.2 mV, respectively; in contrast, the **P-SH** film treated with citrate-capped AuNPs (**AuNPs@P-SH**) had a much more negative surface charge (zeta potential: −17.1 mV). In addition, a less-changed zeta potential appeared for **P** after AuNP treatment, due to no binding sites being available for AuNP immobilization (Figure 4b), consistent with the SEM results of less AuNPs on the surface of P in Figure S2 (see Supplementary Material). Prior to studying the electrochemical responses of all of the OECT devices for the selective sensing of DA in the presence of AA and UA, we used cyclic voltammetry (CV) to examine the electrochemical properties of **P**, **PP-SH**, and **AuNPs@PP-SH** electrodes in 1× PBS buffer (pH 7.4). As displayed in Figure 4c, the experimental cyclic voltammograms of all of the electrodes featured no obvious electrochemical responses at a scan rate of 100 mV s^−1^ when the voltage was swept from −0.8 to +0.8 V in the 1× PBS (pH 7.4) buffer solution. The areas enveloped by the sweep of the CV curves decreased in the order **P** > **PP-SH** > **AuNPs@PP-SH**, revealing that decoration with the citrate-capped AuNPs decreased the negative charge capacity density of the **AuNPs@PP-SH** electrode. Moreover, electrochemical impedance spectroscopy (EIS) revealed a decrease in impedance that followed the order **AuNPs@PP-SH** > **PP-SH** ≈ **P** in the low frequency range from 10^−1^ to 2 × 10^2^ Hz; in contrast, the decrease in impedance followed the order **PP-SH** **≈** **P** > **AuNPs@PP-SH** in the high frequency range from 2 × 10^2^ to 10^5^ Hz (Figure 4d). These experimental results suggested that **AuNPs@PP-SH** was the candidate BEI material for preventing negatively charged AA and UA from reaching the PEDOT electrode, with no obvious loss in ionic conductivity, thereby allowing highly selective detection of DA during electrochemical operation.

### DPV Measurements of the Selective Detection of DA

**Figure 5:**
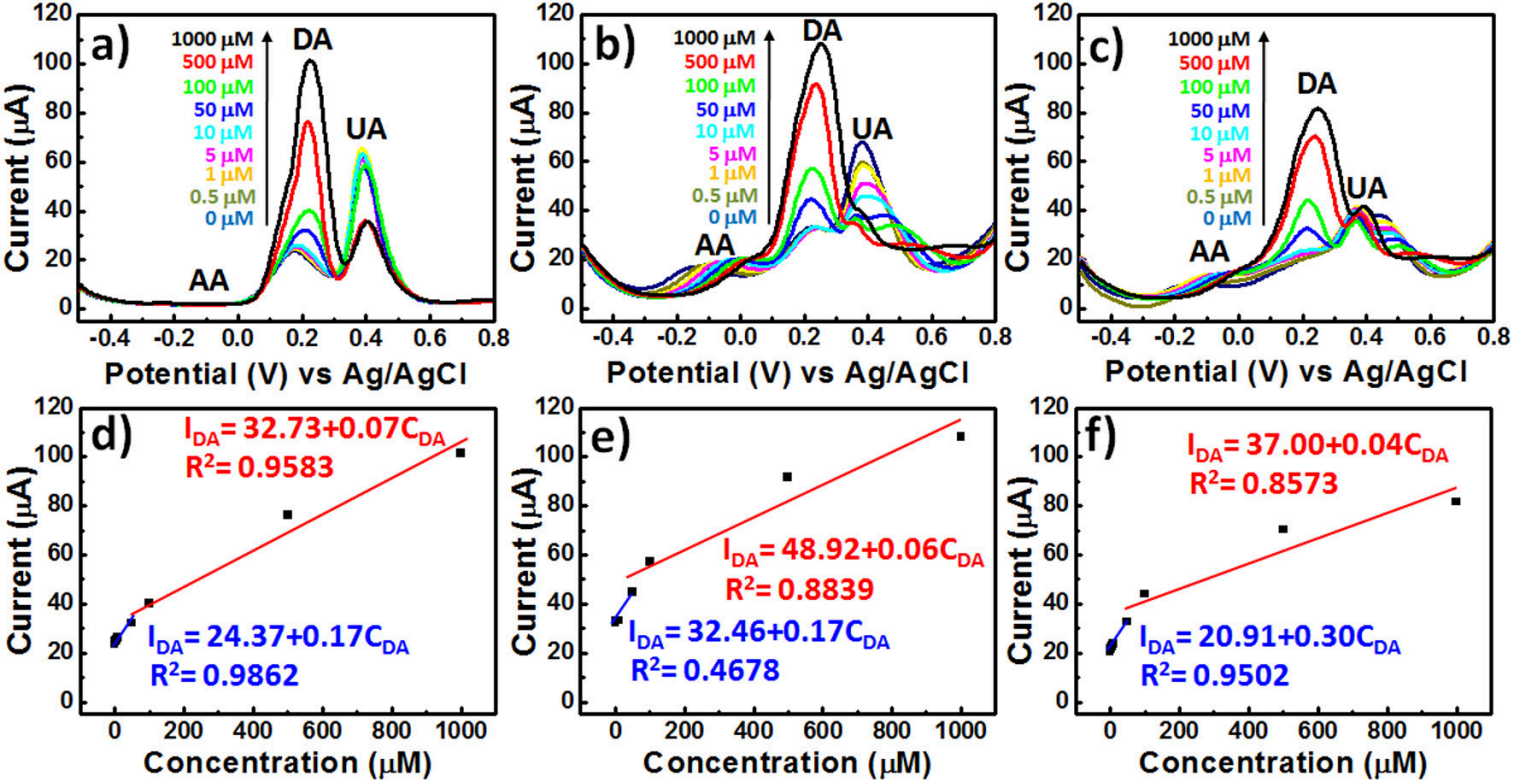
DPV curves of various PEDOT films in 1× PBS (pH 7.4) containing AA, DA, and UA (1 mM AA; 0.5–1000 μM DA; 1 mM UA): **(a) P**, **(b) PP-SH**, and **(c) AuNPs@PP-SH**. **(d–f)** Oxidation DPV peak currents of DA (*I*_DA_) plotted with respect to the DA concentration (*C*_DA_) for **(a–c)**, respectively.

We employed more sensitive differential pulse voltammetry (DPV) measurements to explore the ability of PEDOT-based BEIs to selectively detect DA in the presence of AA and UA (Figures 5a–f). The interference effect of AA at a concentration of 1 mM on all of the PEDOT-based electrodes would be negligible during the determination of DA, because AA caused no obvious changes in the current response; the oxidation peak potential for AA appeared at ~-0.1 V. Figure 5a reveals that the DPV curves of the **P** electrode could be used for the determination of DA at various concentrations (0.5, 1, 5, 10, 50, 100, 500, and 1000 μM), with two linear regions in the plot of the DPV peak current (*I*_DA_) observed for DA oxidation with respect to the DA concentration (*C*_DA_) in the presence of other AA and UA metabolites, the concentrations of which were fixed at 1 mM. The DPV responses obtained from the **P** electrodes was represented by the linear regression function *I*_DA_ = 24.37 + 0.17*C*_DA_ with a correlation coefficient (*R*^2^) of 0.9862 in the low-concentration region of DA, and by the linear regression function *I*_DA_ = 32.73 + 0.07*C*_DA_ with a correlation coefficient (*R*^2^) of 0.9583 in the high-concentration region of DA (Figure 5d). Similar trends—of two slopes in all calibration curves—have appeared in previous reports of DA adsorption on electrode surfaces (Yue et al., 2014; Su et al., 2017). This feature can be explained by considering that a monolayer adsorption would first result in higher sensitivity in the low-concentration region, due to the unsaturated active sites of the PEDOT-based BEIs; multilayer adsorption of analytes would, however, limit the availability of active sites and, therefore, result in decreased sensitivity in the high-concentration region. Unfortunately, the interference effect was much more prominent at lower concentrations of DA, potentially bringing about misjudgment of the UA concentration. Although the **PP-SH** electrode provided a DPV peak current similar to that of the **P** electrode, UA had a greater interference effect, with the correlation coefficient (R^2^ = 0.4678) in the low-concentration region being much lower than that for the **P** electrode (R^2^ = 0.9862) (Figures 5b,e). This observation suggests that the addition of UA might influence the adsorption or oxidation of DA and, therefore, fail to precisely detect DA in the low-concentration region. Notably, however, the DPV curves of the **AuNPs@PP-SH** electrode were not affected by the interference of UA and could be used to precisely determine various DA concentrations, presumably because the high specific area of the citrate-capped AuNPs (presenting carboxyl groups) prevented UA from reaching the electrode surface—in addition to the thiol-functionalized PEDOT materials exhibiting superior electron transfer properties (Ali et al., 2018). The **AuNPs@PP-SH** electrodes also exhibited two linear regression equations for the DA concentrations in the presence of AA and UA (Figure 5f): for low concentrations of DA it was *I*_DA_ = 20.91 + 0.3029*C*_DA_ (R^2^ = 0.9502) with a detection limit of 79 nM (S/N = 3); for high concentrations of DA it was *I*_DA_ = 37.00 + 0.04*C*_DA_ (R^2^ = 0.8573). To prove that this **AuNPs@PP-SH** electrode could also exhibit good selectivity against a broad range of other analytes, we further selected other protein-bound uremic toxins, such as p-cresol (PC), indoxyl sulfate (IS), and hippuric acid (HA), in the preparation of three different kinds of quaternary mixtures containing [PC (1 mM) + DA (1 mM) + UA (1 mM) + AA (1 mM)], [IS (1 mM) + DA (1 mM) + UA (1 mM) + AA (1 mM)], and [HA (1 mM) + DA (1 mM) + UA (1 mM) + AA (1 mM)] in PBS buffer solution and tested them on the chips using the DPV measurement, indicating that there were no additional peaks (originated from PC, IS, and HA) observed in the range from −0.5 to 0.8 V (Figure S3, see Supplementary Material). Therefore, this biosensor have a good selectivity toward DA against a broad range of AA, UA, PC, IS, and HA analytes through the electrochemical approach.

### Output Characteristics and Transfer Curves of OECT/SERS Hybrid Biosensors

**Figure 6:**
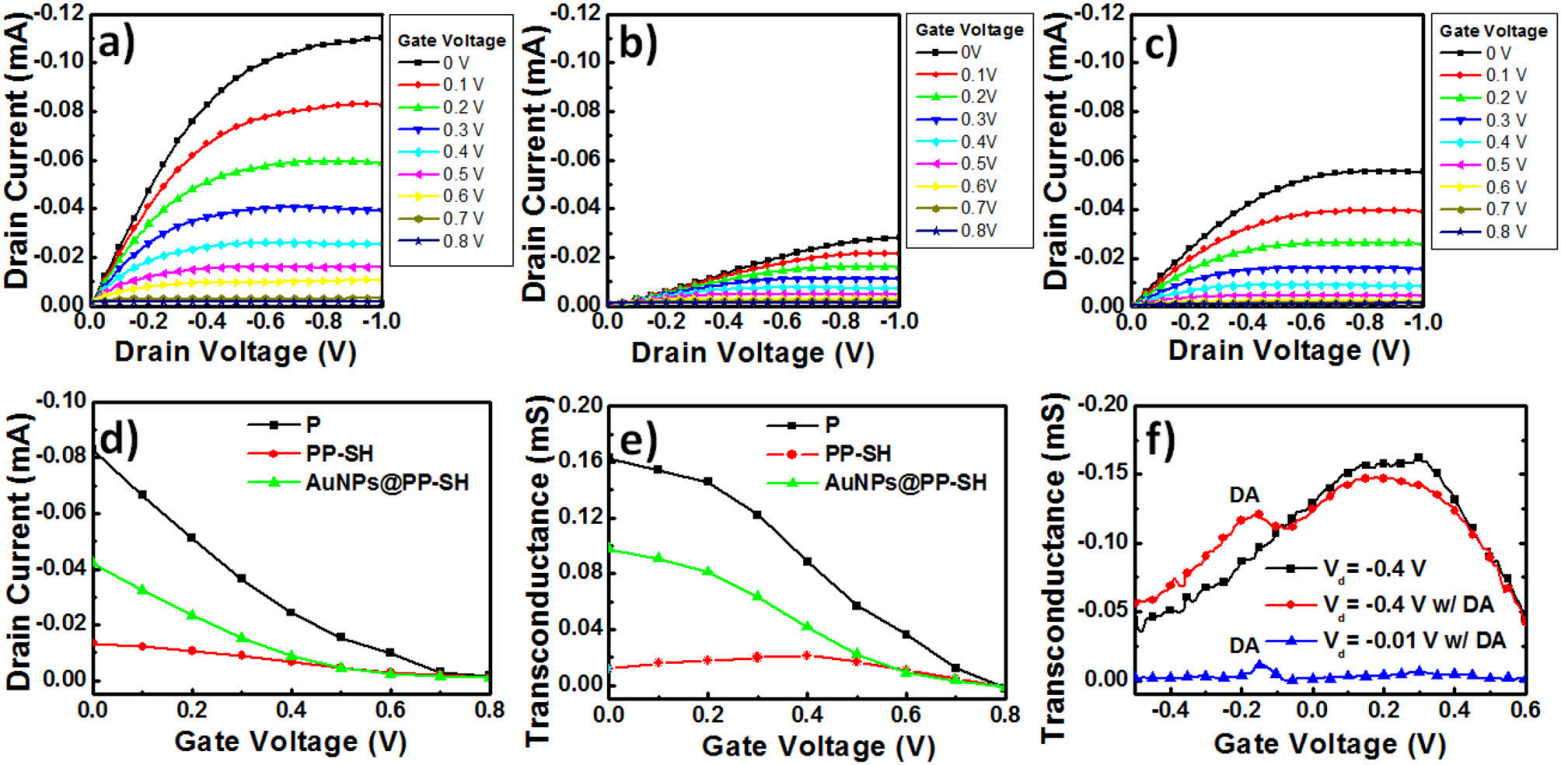
**(a–c)** Output curves for PEDOT-based OECT devices: **(a)** the **P** OECT, **(b)** the **PP-SH** OECT, and **(c)** the **AuNPs@PP-SH** OECT. **(d)** Transfer curves for PEDOT-based OECT devices (**P**, **PP-SH**, and **AuNPs@PP-SH** OECTs). **(e)** Transconductance plotted with respect to *V*_g_ for PEDOT-based OECT devices (**P**, **PP-SH**, and **AuNPs@PP-SH** OECTs): potential swept from 0 to 0.8 V. **(f)** Transconductance plotted with respect to *V*_g_ for **AuNPs@PP-SH** OECTs, and recorded in 1× PBS (pH 7.4) with 1 mM DA at various values of *V*_d_: potential swept from −0.5 to +0.6 V.

To explore the enhancement effect of the AuNPs on the OECT device performance, we measured the *I*_d_-*V*_d_ output characteristics, *I*_d_-*V*_g_ transfer curves, and *g*_m_-*V*_g_ curves to determine the differences in the electrical conductivities and electrochemical properties of the various PEDOT-based active layer channels (featuring the **P**, **PP-SH**, and **AuNPs@PP-SH** films) at the level of realistic devices (Figure 6). Because the transconductance (*g*_m_) depends strongly on the channel geometry, it is necessary to compensate for the device geometries (channel length, channel width, film thickness) when comparing the performances of different OECT channel materials. It has been studied that the OECT transconductance of PEDOT:PSS-based devices at saturation is proportional to (*W*×*d*/*L*), where the channel geometry of PEDOT:PSS thin film was designed in OECTs [width (*W*); length (*L*); thickness (*d*)] (Rivnay et al., 2015). More importantly, the previous report suggested that the use of large-area thin film would result in maximizing the signal-to-noise (SNR) for biochemical sensing applications (Stoop et al., 2017). Therefore, in this study, we have used three different thicknesses (100, 200, and 400 nm) of PEDOT:PSS thin films [width (*W*): 1.5 mm; length (*L*): 5 mm] to study the effect of membrane thickness on the **P** OECT behaviors. Although the 400 nm-thickness sample exhibited higher transconductance than others, a higher turn-off gate voltage (~0.8 V) and a shifted transconductance peak (~0.35 V) were also obtained, thereby faring away from the sweeping potential range of DA (between −0.5 and +0.6 V) (Figure S4, see Supplementary Material). Accordingly, taking the factors influencing device performance together, it can be concluded that the 200 nm-thickness of **P** underlying layer displayed the optimized OECT performance and could possibly be used for DA detection in the presence of AA and UA. For this study, we controlled the thickness of the **P**, **PP-SH**, and **AuNPs@PP-SH** active layer channels of the OECTs at ~290 nm (through spin-coating) and confined the same channel length and width (through the use of a CO_2_ laser engraving system). For example, the thickness of the PEDOT:PSS film for **P** device was approximately 290 nm; the thicknesses of the PEDOT:PSS underlying layer (**P**) and the thiol-functionalized PEDOT top layer (**P-SH**) were ~200 and 90 nm, respectively, in both the **PP-SH** and **AuNPs@PP-SH** devices. Figures 6a–c present the output characteristics of OECTs prepared with **P**, **PP-SH**, and **AuNPs@PP-SH** as active layer channels, measured under a negative sweeping bias (from 0 to −1.0 V) on the drain and stepped gate voltages varied from 0 to 0.8 V (using the Ag/AgCl gate electrode). Briefly, all of the devices, featuring the three different kinds of active layers, exhibited the depletion regime of an OECT under typical low-voltage operation, with the drain current decreasing as the applied gate voltage increased. A comparison of the electrical conductivities and output characteristics of the three different kinds of active layers in the OECTs (Figures 3f, 6), revealed that the drain current of the OECTs was strongly correlated to the electrical conductivity of the PEDOT-based active layer and the electrocatalytic activity of the AuNP coating. For example, when the values of *V*_g_ and *V*_d_ were fixed at 0 and −1 V, respectively, all of the channels exhibited their highest drain currents; a decrease in the on-current was found, however, to follow the order **AuNPs@PP-SH** > **PP-SH**, suggesting that the high specific area of the AuNP coating may have enhanced the electrical conductivity of **PP-SH**; a decrease in the off-current followed the order **P** > **AuNPs@PP-SH**, suggesting that the AuNP-decorated **PP-SH** channel could boost the electrochemical process in the active layer channels to switch the transistor off efficiently, according to the de-doping phenomenon in the channel (Figure 6d).

OECT devices can amplify an input signal and lead to an increase in current modulation in the presence of analyte oxidation; therefore, they might be represent excellent candidates for biosensing. In particular, a pivotal parameter for OECTs is the transconductance (*g*_m_), which can be obtained from the derivative of the *I*_d_-*V*_g_ transfer curve (*g*_m_ = ∂*I*_d_/∂*V*_g_) (Figure 6d). When applying a constant value of *V*_d_ (−0.4 V) and varying the value of *V*_g_ (from 0 to 0.8 V) applied to the OECTs, the resulting *g*_m_-*V*_g_ curves of the **P**, **PP-SH**, and **AuNPs@PP-SH** devices provided their maximum values of *g*_m_ near values of *V*_*g*_ of 0, 0, and 0.4 V, respectively (Figure 6e). The experimentally measured transconductances plotted with respect to *V*_g_ in the range from −0.5 to +0.6 V confirmed the background signal of the **AuNPs@PP-SH** OECTs, as well as the location of the DA signal in the *g*_m_-*V*_g_ curves (Figure 6f). The **AuNPs@PP-SH** OECTs operated at a value of *V*_d_ of −0.4 V would result in a broad background signal with a maximum value of *g*_m_ located near 0.2 V, with an additional peak appearing near a gate voltage of −0.15 V in the presence of DA (1 mM) in 1× PBS (pH 7.4) buffer solution. Notably, operating the OECT at a value of *V*_d_ of −0.01 V minimized the background signal of the PEDOT film in the **AuNPs@PP-SH** device, potentially providing a more accurate determination of DA.

### Response of OECTs to the Addition of DA

**Figure 7:**
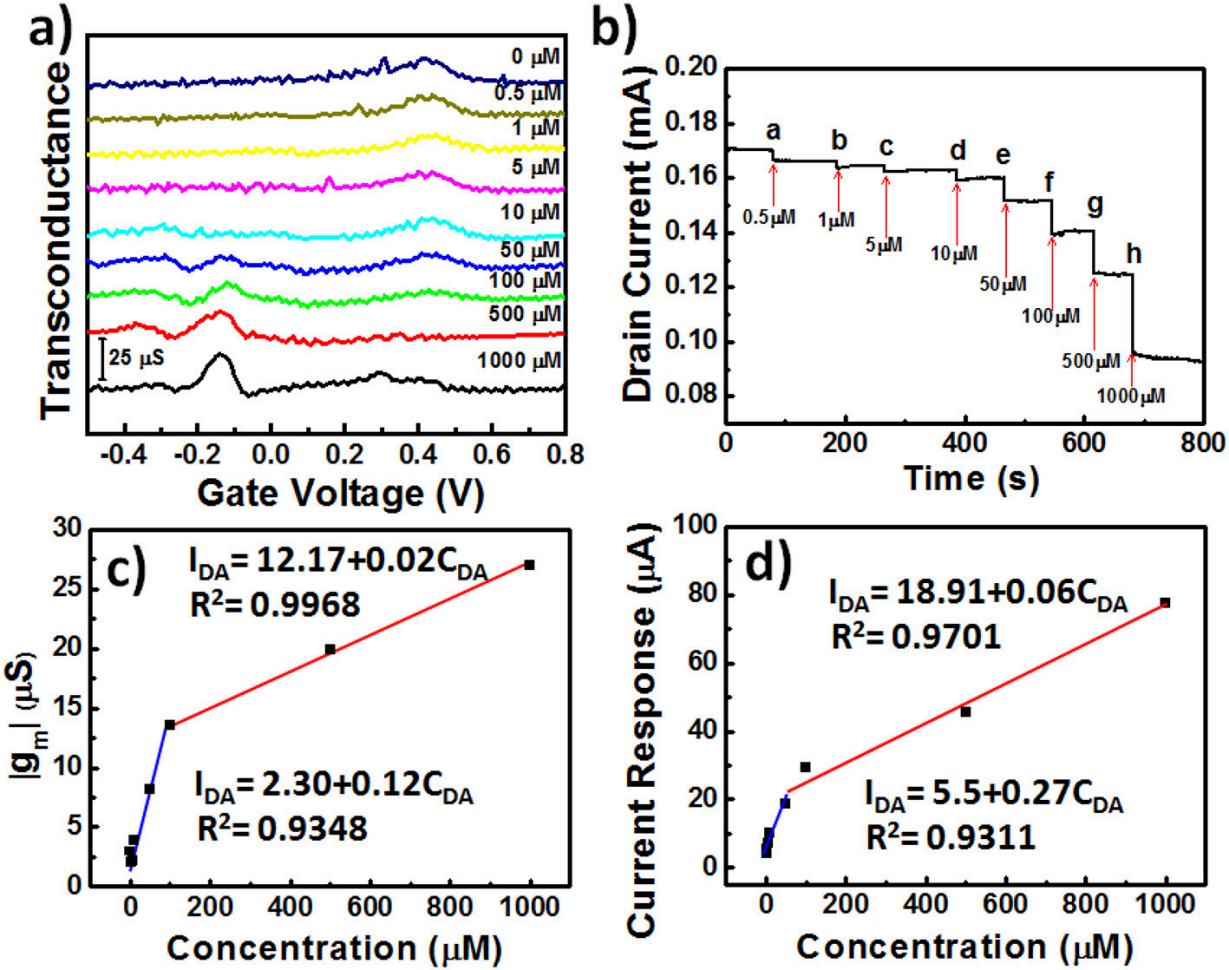
**(a)** Transconductance (*g*_m_) plotted with respect to *V*_g_ for **AuNPs@PP-SH** OECTs, and recorded in 1× PBS (pH 7.4) containing AA, DA, and UA (1 mM AA; 0.5–1000 μM DA; 1 mM UA) at a value of *V*_d_ of −0.01 V: potential swept from −0.5 to +0.8 V. **(b)** Amperometric response (*I*_d_ plotted with respect to time) recorded with incremental additions of DA in the presence of 1 mM AA and 1 mM UA. **(c)** Transconductance (*g*_m_) plotted with respect to DA concentration (*C*_DA_) for **(a)**. **(d)** Drain current response plotted with respect to DA concentration (*C*_DA_) for **(b)**.

Our goal for this study was to demonstrate that the developed **AuNPs@PP-SH** OECTs could be employed for the highly selective determination of DA in the presence of AA and UA as interfering agents. Figure 7a displays the transconductance (*g*_m_) responses of **AuNPs@PP-SH** OECTs measured at various DA concentrations (0, 0.5, 1, 10, 50, 100, 500, and 1000 μM) in 1× PBS (pH 7.4) buffer solution, in the presence of AA and UA, at a constant value of *V*_d_ of −0.01 V. The generated Faradic current appears to be the main working mechanism underlying the effect of DA electro-oxidation on the gate electrode upon increasing the concentration of DA. Therefore, the transfer characteristic curves shifted to more negative gate voltages, due to the increase in the effective gate voltage applied on the transistor; the greater values of *g*_m_ were due to the decrease in the potential drop at the electrolyte–gate interface (Gualandi et al., 2018). For these measurements, the **AuNPs@PP-SH** OECT provided sensitivity similar to that of the DPV method (Figure 5f) for the determination of DA, with less interference from AA and UA. The **AuNPs@PP-SH** devices functioned with two linear regression equations for the determination of the DA concentrations (Figure 7c): for low concentrations of DA it was *I*_DA_ = 2.30 + 0.12*C*_DA_ (*R*^2^ = 0.9348) with a detection limit of ~33 nM (S/N = 3); for high concentrations of DA, it was *I*_DA_ = 12.96 + 0.01*C*_DA_ (*R*^2^ = 0.9987). Gualandi et al. (2018) found that a maximum amplification of PEDOT:PSS OECTs operated at a value of *V*_g_ close to 0 V would provide higher sensitivity for biosensing, while values of *V*_g_ lower than 0 V would result in higher electrochemical potentials for the channels and higher rates of hole regeneration, providing higher rates of charge-transfer for real-time monitoring of targeted analyte concentrations. Therefore, for our present measurements, we applied values of *V*_d_ and *V*_g_ of −0.4 and −0.01 V, respectively, to the **AuNPs@PP-SH** OECTs to ensure detections with high sensitivity and rapid responses, and measured the amperometric response of the drain current (*I*_d_-time curve) upon the continuous addition of DA (Figure 7b). When greater degrees of DA oxidation occurred in the **AuNPs@PP-SH** active layer channel, it consumed more cationic species and, thereby, led to a decrease in the value of *I*_d_, characterized by two empirical equations (Figure 7d): a linear regression equation for low concentrations (0.5–100 μM) of DA of *I*_DA_ = 5.5 + 0.27*C*_DA_ (*R*^2^ = 0.9311) with a detection limit (S/N = 3) of 37 nM; for high concentrations (100–1000 μM) of DA, the corresponding linear regression equation was *I*_DA_ = 18.91 + 0.06*C*_DA_ (*R*^2^ = 0.9701).

### SERS Experiments for Selective Detection of PC

**Figure 8:**
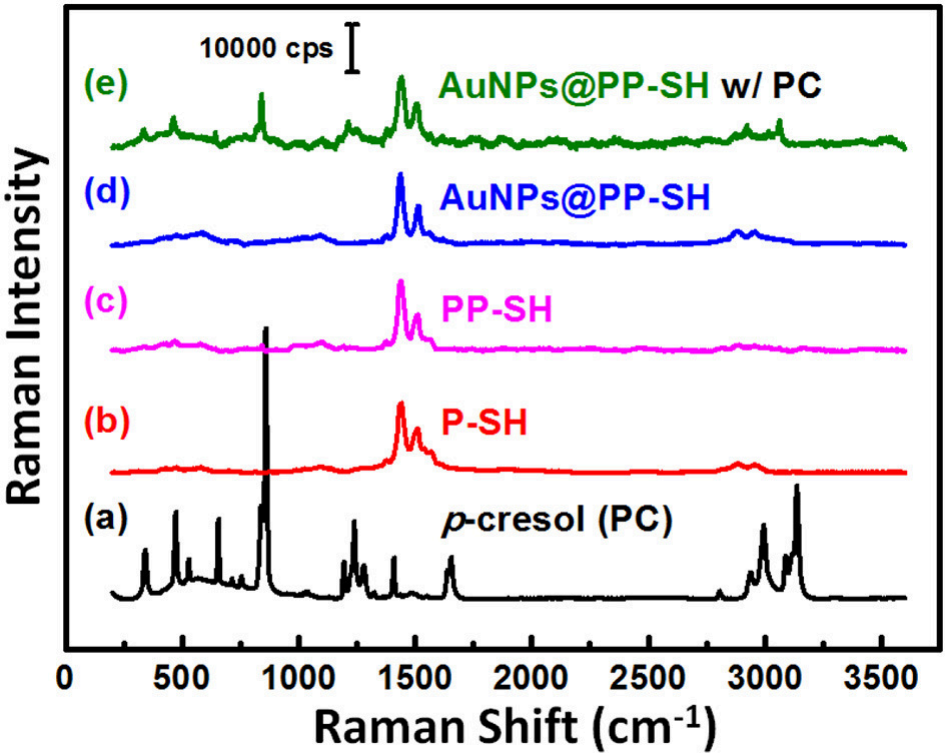
**(a–d)** Raman spectra of PC, **P-SH**, **PP-SH**, and **AuNPs@PP-SH**. **(e)** SERS spectrum of the **AuNPs@PP-SH** device in the presence of 1 mM PC, recorded at an excitation laser wavelength of 633 nm.

**Figure 9:**
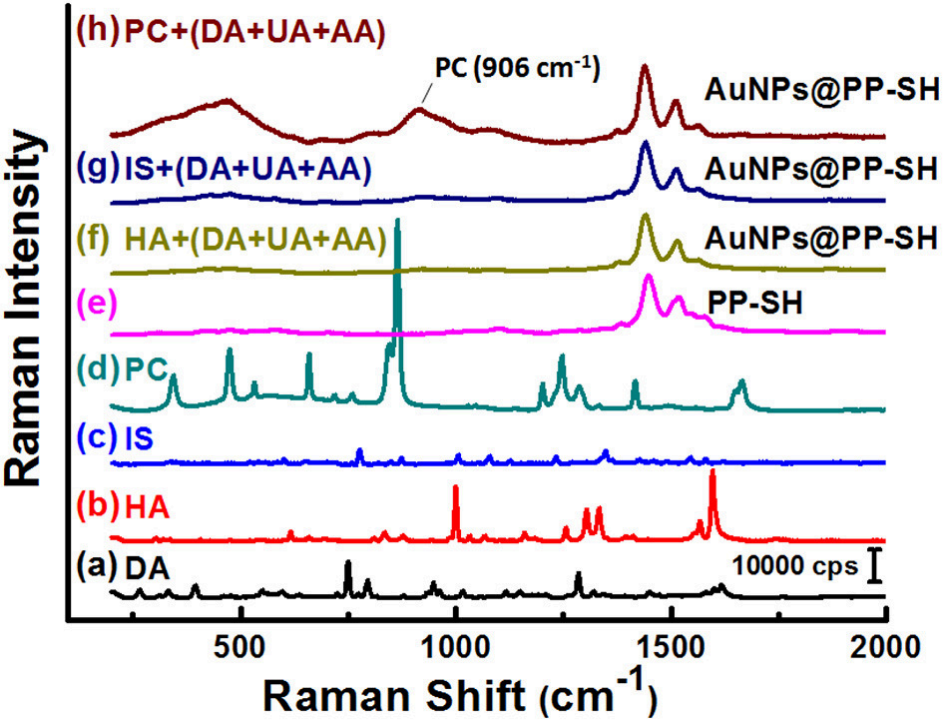
**(a–e)** Raman spectra of DA, HA, IS, PC, and **PP-SH**. SERS spectra of the **AuNPs@PP-SH** device, recorded in 1× PBS (pH 7.4) in the presence of a quaternary mixture containing **(f)** HA (1 mM), DA (1 mM), UA (1 mM), and AA (1 mM); **(g)** IS (1 mM), DA (1 mM), UA (1 mM), and AA (1 mM); **(h)** PC (1 mM), DA (1 mM), UA (1 mM), and AA (1 mM) at an excitation laser wavelength of 633 nm.

To explore the possibility of using the same OECT device for the detection of PC in biological fluids through SERS spectroscopy, we recorded the Raman spectra of liquid-phase pure PC and studied its SERS spectra recorded on the **PP-SH** and **AuNPs@PP-SH** devices (Figure 8). Very strong Raman bands of pure PC appear near 850 and 830 cm^−1^, assigned to the canonical Fermi doublet (Takahashi and Noguchi, 2007) (Figure 8a). As displayed in Figures 8b–d, the Raman fingerprints of the **P-SH**, **PP-SH**, and **AuNPs@PP-SH** devices were similar to those previous reported for commercial PEDOT:PSS materials (Wang et al., 2018b), with the vibration peaks of PEDOT at 1374, 1437, and 1513 cm^−1^ assigned to C_β_-C_β_ stretching, C_α_ = C_β_ symmetrical, and C_α_ = C_β_ symmetrical vibrations, respectively. The Raman signal at 860 cm^−1^ from the liquid-phase PC appeared for our **AuNPs@PP-SH** device; therefore, it could be used for the quantitative detection of PC (Figure 8e). To evaluate the SERS enhancement effect of the **AuNPs@PP-SH** devices for the detection of PC in aqueous fluids, we prepared a quaternary mixture containing PC (1 mM), DA (1 mM), UA (1 mM), and AA (1 mM) in PBS buffer solution and tested it on the chips using SERS spectroscopy with comparison to the mixture solutions of [HA (1 mM) + DA (1mM) + UA (1 mM) + AA (1 mM)] and [IS (1 mM) + DA (1mM) + UA (1 mM) + AA (1 mM)] (Figure 9). Previous reports have shown that the strongest peak at 1331, 1386, and 1567 cm^−1^ can be chosen to estimate the correlation between the Raman intensity and the concentration of DA, AA, and UA, respectively (Pucetaite et al., 2016; Cholula-Díaz et al., 2018; Phung et al., 2018). The **AuNPs@PP-SH** devices provided a higher-intensity Raman signal for PC, due to its greater SERS activity (SERS hot spots), in the presence of DA, UA, and AA than did the **PP-SH** devices; nevertheless, discrimination of the overlapping region of signals from the DA, AA, and UA analytes from the background signals of **AuNPs@PP-SH** devices remained a major challenge in the fingerprint region (1300–1600 cm^−1^) (Figure 9h).

**Figure 10:**
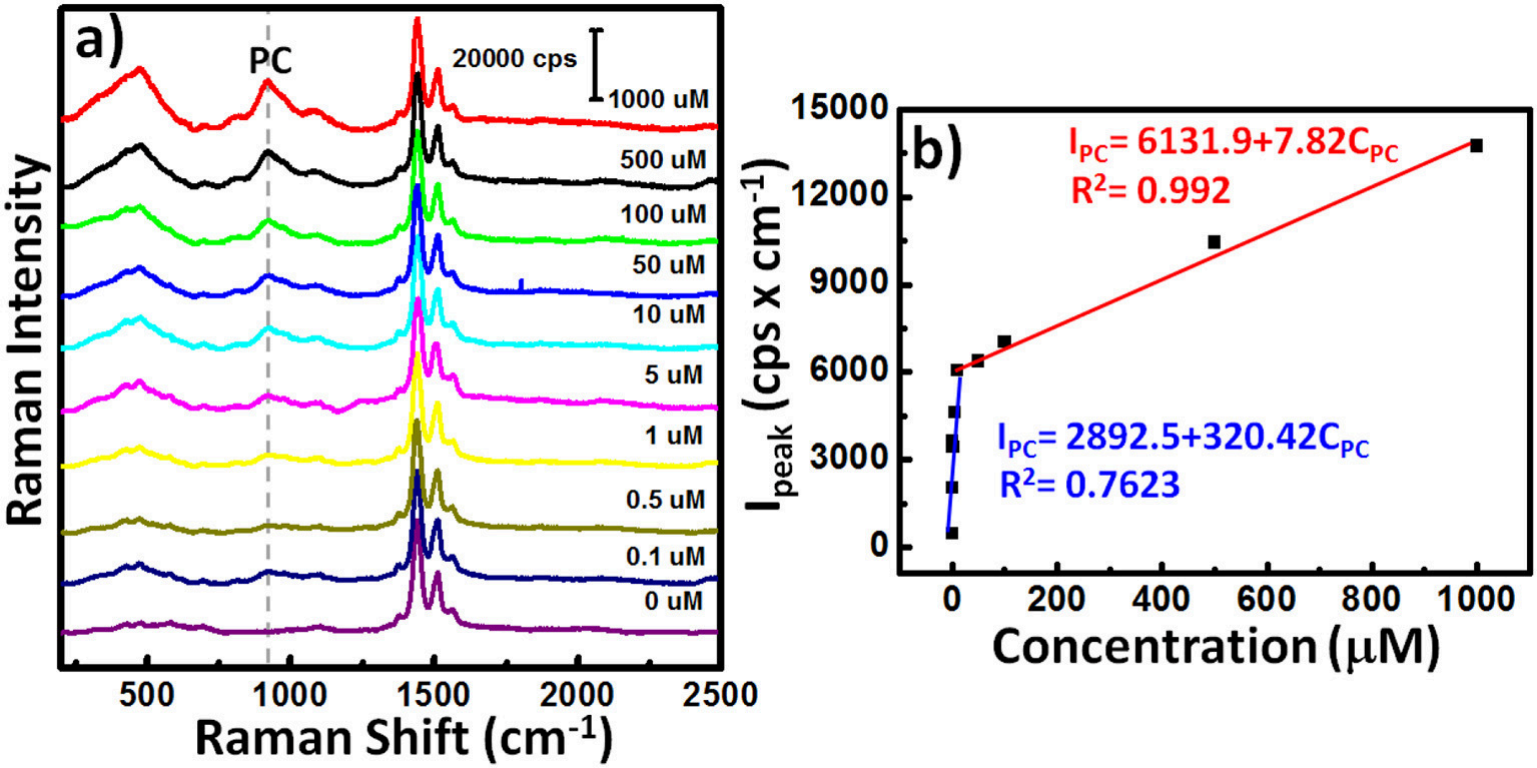
**(a)** SERS spectra of the **AuNPs@PP-SH** device in the presence of various concentrations of PC and 1 mM DA, 1 mM AA, and 1 mM UA. **(b)** SERS signal intensity (*I*_PC_) at 906 cm^−1^ plotted with respect to PC concentration (*C*_PC_) for **(a)**.

Because the serum concentrations of PC in healthy control and hemodialysis patients are 8.6 ± 3.0 and 88.7 ± 49.3 μM (De Smet et al., 1998), we varied the concentration of PC (*C*_PC_) in a predictable manner (0.1, 0.5, 1, 5, 10, 50, 100, 500, and 1000 μM) and then used our **AuNPs@PP-SH** device to quantify the SERS signal responses in the presence of 1 mM DA, 1 mM UA, and 1 mM AA (Figure 10a). The SERS intensity (*I*_PC_) of the band at 906 cm^−1^ (assigned to a large red-shift of the canonical Fermi doublet) could be expressed quantitatively using two empirical equations (Figure 10b): for low concentrations (0.1–50 μM) of PC it was *I*_PC_ = 2892.5 + 320.42*C*_PC_ (*R*^2^ = 0.7623); for high concentrations (50–1000 μM) of PC it was *I*_PC_ = 6131.9 + 7.82*C*_PC_ (*R*^2^ = 0.992). The sensitivity of SERS in the region of high PC concentration (50–1000 μM) was much lower than that in the region of low PC concentration (0.1–50 μM) because of the hotspot effect decreases dramatically—indeed, exponentially—with the distance between the SERS-active substrates. In total, all of these SERS-active demonstrations have reveal that the **AuNPs@PP-SH** device was capable of detecting PC over a wide concentration range, with high specificity when using a ternary aqueous solution of DA, UA, and AA.

## Conclusion

We have developed a PEDOT-based 3D-OECT device decorated with a high density of AuNPs for the effective detection of DA in the presence of multiple interferers, and as SERS-active substrates for the selective detection of PC on the same chip. This novel OECT/SERS hybrid biosensor platform featured a highly conductive PEDOT:PSS film as the underlying layer, which greatly enhanced the transconductance and sensitivity of the OECTs for the electrochemical sensing of DA in the presence of AA and UA, and provided an amperometric response to DA with a detection limit of 37 nM in the linear range 50 nM−100 μM. The AuNP-immobilized thiol-functionalized PEDOT film as the top interfacial layer not only improved the performance of the OECTs for the selective detection of DA, due to the signal amplification effect of AuNPs with high catalytic activity, but also enabled downstream detection of PC in biological fluids, due to the outstanding enhancement effect of SERS hotspots. Such OECT/SERS hybrid biosensors decorated with high densities of AuNPs display new versatility, encouraging the design of next-generation biosensors for point-of-care diagnostics.

## Author Contributions

P-CH and C-LC contributed to the fabrication of the OECT/SERS hybrid biosensors, as well as to the DPV and OECT measurements. C-HL and Y-HT contributed to the synthesis of EDOT-SH. J-AC performed the experiments involving SERS detection. C-KW, C-WK, HY, and PC supervised the experiments, critically reviewed and edited the manuscript. Y-SH contributed to the experimental design and wrote the main manuscript text. All authors have reviewed the manuscript and approved its final version.

### Conflict of Interest Statement

The authors declare that the research was conducted in the absence of any commercial or financial relationships that could be construed as a potential conflict of interest.
