# Supplementary material for: Organic Electrochemical Transistors/SERS-Active Hybrid Biosensors Featuring Gold Nanoparticles Immobilized on Thiol-Functionalized PEDOT Films
Source: Front Chem. 2019 Apr 26;7:281. doi: 10.3389/fchem.2019.00281 (PMC6498878; doi:10.3389/fchem.2019.00281)
Supplement: Supplementary file 1 [file Table_1.doc]

Electronic Supporting Information

**Organic electrochemical transistors/SERS-active hybrid biosensors featuring gold nanoparticles immobilized on thiol-functionalized PEDOT films**

Jia-An Chou^a,+^, Chieh-Lin Chung^a,+^, Po-Cheng Ho^a,b,+^, Chun-Hao Luo^b^, Yu-Han Tsai^b^, Chung-Kuan Wu^c^, Chiung-Wen Kuo^d^, Yu-Sheng Hsiao^a,*^, Hsiao-hua Yu^b,*^, and Peilin Chen^d,*^

^a^ Department of Materials Engineering, Ming Chi University of Technology, New Taipei City 24301, Taiwan.

E-mail: [yshsiao@mail.mcut.edu.tw](mailto:yshsiao@mail.mcut.edu.tw); Fax: +886-2-2908-4091

^b^ Institute of Chemistry, Academia Sinica, Taipei 11529, Taiwan.

E-mail: [bruceyu@gate.sinica.edu.tw](mailto:peilin@gate.sinica.edu.tw); Fax: +886-2-2783-1237

^c^ Division of Nephrology, Department of Internal Medicine, Shin-Kong Wu Ho-Su Memorial Hospital, Taipei 11101, Taiwan.

^d^ Research Center for Applied Sciences, Academia Sinica, Taipei 11529, Taiwan.

E-mail: [peilin@gate.sinica.edu.tw](mailto:peilin@gate.sinica.edu.tw); Fax: +886-2-2782-6680

^+^These authors contributed equally to this work.

**1. EDOT Monomers Synthesis**

The EDOT-SH monomer was prepared in three steps from EDOT-OH: to EDOT-Br, EDOT-SAc, and EDOT-SH (**Scheme S1**). EDOT-Br, EDOT-SAc, and EDOT-SH were synthesized using the following procedures, according to a previous literature report (Goda, T., Toya, M., Matsumoto, A., and Miyahara, Y. (2015). Poly (3,4-ethylenedioxythiophene) bearing phosphorylcholine groups for metal-free, antibody-free, and low-impedance biosensors specific for C-reactive protein. *ACS Appl. Mater. Interfaces*, 7, 27440–27448).

**EDOT-Br (1).** EDOT-OH (5.00 g, 29.0 mmol) and CBr_4_ (14.4 g, 43.6 mmol) were dissolved with vigorous stirring in THF (35 mL) in a 100-mL round-bottom flask. The flask was cooled in an ice-bath and then PPh_3_ (9.14 g, 34.8 mmol) was added. The mixture was stirred overnight at room temperature under N_2_. After the starting materials had been consumed, the THF was evaporated under reduced pressure. The residue was partitioned between EtOAc and 1 N HCl; the combined organic phases were dried (MgSO_4_) and concentrated under reduced pressure. The residue was purified through column chromatography (EtOAc/hexane, 1:9) to yield a white solid (5.38 g, 79.2%).

**EDOT-SAc (2).** K_2_CO_3_ (31.8 g, 230 mmol) was added with vigorous stirring to a solution of EDOT-Br (5.38 g, 23.0 mmol) in acetone. KSAc (3.94 g, 34.5 mmol) was added and then the mixture was stirred at room temperature overnight under N_2_. After the reaction had reached completion, the K_2_CO_3_ was filtered off and the solvent was evaporated under reduced pressure. The residue was partitioned between 1 N HCl and EtOAc; the combined organic phases were dried (MgSO_4_) and concentrated under reduced pressure. The residue was purified through short column chromatography (EtOAc/hexane, 1:4) to yield a yellowish oil (4.17 g, 78.8%).

**EDOT-SH (3).** NaOMe (0.98 g, 18.1 mmol) was added to a solution of EDOT-SAc (4.17 g, 18.1 mmol) in MeOH and then the mixture was stirred under N_2_ at room temperature for 2–3 h (monitored through TLC). After the starting materials had been consumed, the reaction was quenched through the addition of Amberlite IR-120 resin and then the mixture was filtered. The solvent was evaporated under reduced pressure; the residue was partitioned between EtOAc and 1 N HCl; the combined organic phases were washed with brine, dried (MgSO_4_), and concentrated. The residue was purified through column chromatography (EtOAc/hexane, 1:4) to yield a yellowish stenchy oil (2.83 g, 83.0%). ^1^H NMR (500 MHz, DMSO-*d*_6_, 25 °C): δ 6.58 (s, 2H), 4.31 (d, 1H), 4.16–4.21 (m, 1H), 2.79 (s, 1H), 2.76 (d, 1H), 2.64 (d, 1H). ^13^C NMR (75 MHz, DMSO-*d*_6_): δ 141.22, 99.99, 99.90, 99.75, 74.39, 71.94, 71.91, 66.47, 66.28, 23.74.


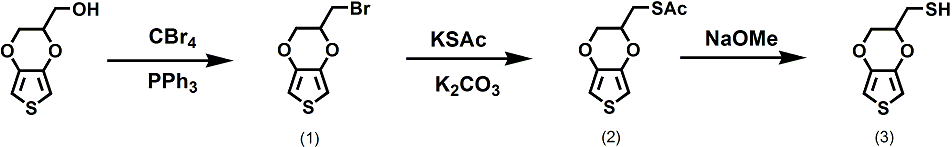


**Scheme S1.** Synthesis of EDOT-SH.

**2. Organic Electrochemical Transistor (OECT) Fabrication**

*Bilayer PEDOT Film Fabrication:* The active layer channels of the OECT devices were prepared through a sequence of coating on patterned ITO glasses, including a high-conductivity PEDOT:PSS underlying layer and a nanostructured thiol-functionalized PEDOT upper layer presenting AuNPs on top (see **Figure 1**). Prior to device fabrication, all of the patterned ITO glasses (<7 Ω sq^–1^) were cleaned sequentially with detergent, DI water, acetone, and isopropyl alcohol. The ITO substrates were then treated with air-plasma for 15 min at 18 W in low-pressure (0.2 mbar) residual air (Harrick Plasma Cleaner, PDC-32G, Harrick Scientific, NY). To fabricate the high-conductivity PEDOT:PSS film (**P**) at a thickness of approximately 290 nm (see **Figures 1b** and **1c**), a mixture of PEDOT:PSS (Clevios PH1000), DMSO (5 wt%), and GOPS (1 wt%) was prepared through sonication (>20 min) and then filtered through a 0.8-μm filter prior to spin-coating (3000 rpm, 60 s) onto cleaned patterned ITO glass substrates, followed by annealing at 130 °C for 1 h. It has been reported that, PEDOT and PSS form globular gel particles with two size distribution peaks in water for the Clevios PH1000 solution; the majority (90%) has a size distribution of 160−940 nm with the maximum at 463 nm, and the average size is 486 nm; the minority (10%) has a size distribution of 16−51 nm, and the average size is 30 nm [1]. The nanostructured thiol-functionalized PEDOT (**P-SH**) upper layer was obtained through chemical oxidative polymerization of the EDOT-SH monomers (see **Figures 1a** and **1c**). The fabrication of the **P-SH** thin film has been described in detail previously (Hsiao, Y.-S., Luo, S.-C., Hou, S., Zhu, B., Sekine, J., Kuo, C.-W., Chueh, D.-Y., Yu, H.-H., Tseng, H.-R., and Chen, P. (2014). 3D Bioelectronic Interface: Capturing Circulating Tumor Cells onto Conducting Polymer‐Based Micro/Nanorod Arrays with Chemical and Topographical Control. *Small* 10, 3012–3017). Solutions of IM (1.47 M; inhibitor) and Fe(III)TOS (2.56 M; oxidizing agent) were prepared in MeOH in separate flasks at 60 °C. The PEDOT precursor was prepared by mixing a separate vial containing EDOT-SH (67 mg) with the IM solution (1.8 mL) and the Fe(III)TOS solution (1.8 mL). For chemical oxidative polymerization of the PEDOT-SH thin film at a thickness of approximately 90 nm, the mixture was spin-coated (2000 rpm, 30 s) onto the **P** sample and then placed directly on a hotplate (105 °C) for 5 min with a covered glass Petri dish. Herein, the bilayer PEDOT film coating on the patterned ITO glass is denoted as **P-SH**.

**3. Materials Characterization**

A field emission-scanning electron microscope (FE-SEM, JEOLJSM 6701F) was used (operated at 10 keV) to examine the surface morphologies of the PEDOT films (**P**, **P-SH**, **AuNPs@P-SH**). The samples for SEM analysis were sputter-coated with platinum (<3 nm). The morphologies of the PEDOT films were analyzed through atomic force microscopy (AFM), using a Bruker Dimension Edge microscope operated in the dynamic force mode at ambient temperature. Transmission electron microscopy (TEM) was performed using a JEOL 2010 microscope (operated at an accelerating voltage of 200 kV) to examine the morphology of the AuNPs. UV–Vis absorption spectra of the aqueous AuNP solutions were recorded using a UV–Vis spectrophotometer (JASCO, V650, Japan) in the wavelength range from 400 to 800 nm. The topographical curves of PEDOT films were measured using a Surfcorder ET200 surface profiler (Kosaka Laboratory, Japan) to obtain the thickness. The electrical conductivities of the PEDOT films were measured using a Keithley 2400 apparatus and the standard four-point probe method. Electrokinetical analysis (Zeta potential) of PEDOT films was performed using a SurPASS electrokinetic analyzer (Anton Paar, Australia); the measurements were performed four times at room temperature, using a streaming current method with 0.001 M KCl as the electrolyte solution; the pH was adjusted to 7.4 by adding either 0.05 M HCl or 0.05 M NaOH. A potentiostat/galvanostat (PGSTAT204, Autolab, Eco Chemie, Netherlands) and a frequency response analysis (FRA) module were used for cyclic voltammetry (CV), electrochemical impedance spectroscopy (EIS), and differential pulse voltammetry (DPV) at room temperature. Phosphate-buffered saline (1× PBS; pH 7.4) was used as the electrolyte in a typical three-electrode cell configuration, which also featured a counter electrode (platinum wire) and a reference electrode [Ag/AgCl (saturated KCl) electrode (RE-1C, BAs)]. An ac sinusoidal signal of 10 mV amplitude was used to obtain the EIS spectra of the samples within a logarithmic frequency range from 10^–1^ to 10^5^ Hz. For CV measurements, the samples were recorded in the potential range from –0.8 to +0.8 V vs Ag/AgCl at a scanning rate of 100 mV s^–1^. For DPV measurements, the following optimized conditions were used to determine the individual concentrations of DA: pulse amplitude: 0.025 V; increment: 0.005 V; sample width: 0.05 s; pulse width: 0.25 s; pulse period: 0.5 s.

**
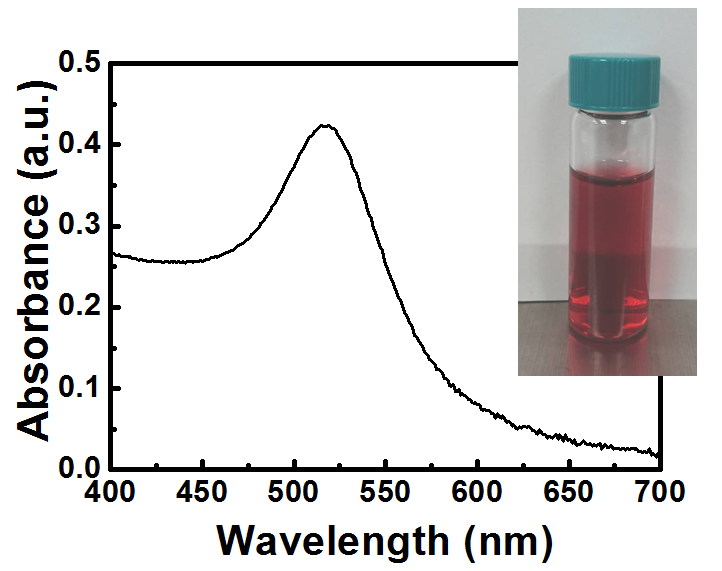
**

**Figure S1.** UV‒Vis absorption spectrum and photograph (inset) of the aqueous solution of citrate-capped AuNPs.


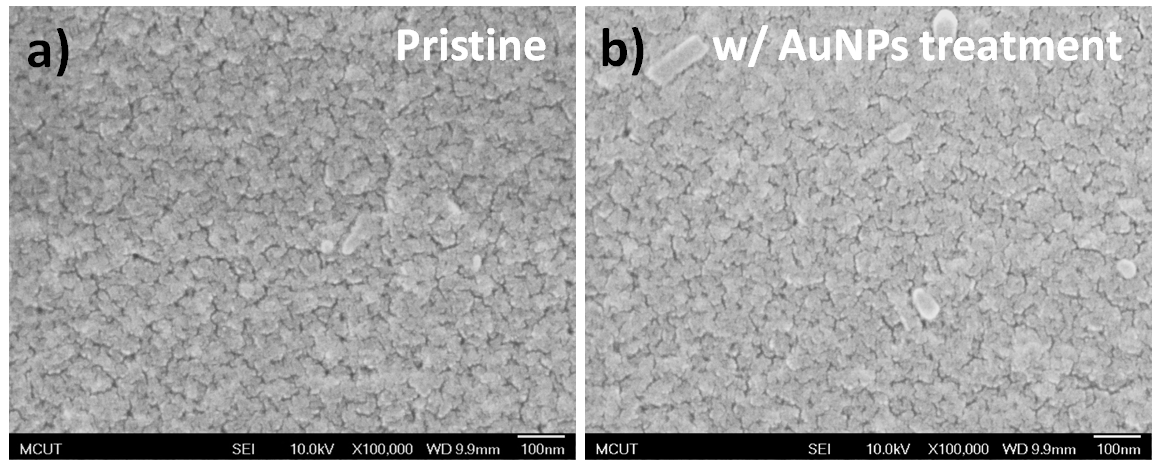


**Figure S2.** SEM images of (a) the pristine **P** film and (b) the **P** film after treatment with the aqueous solution of citrate-capped AuNPs.


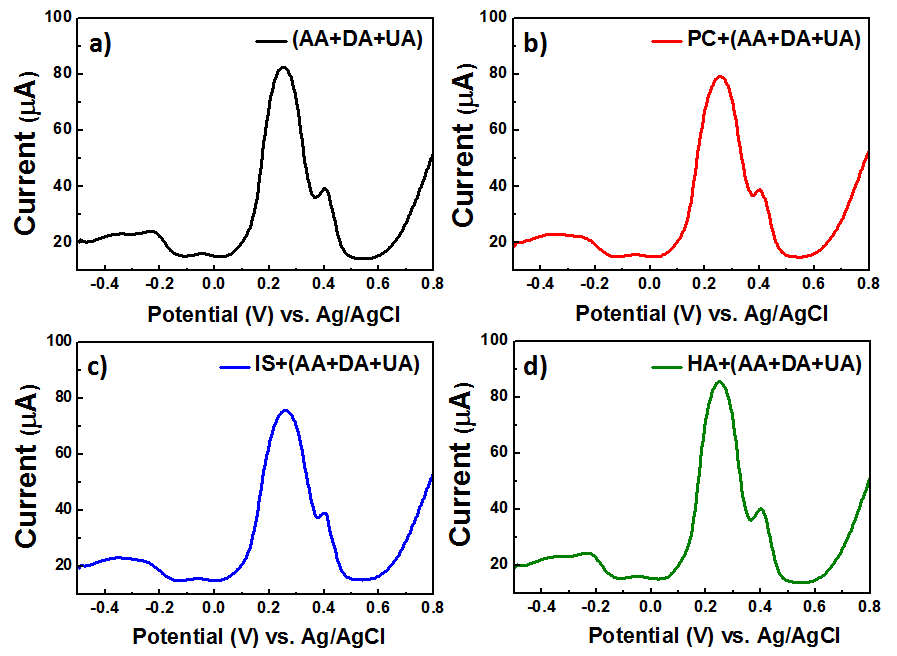


**Figure S3.** DPV curves of **AuNPs@PP-SH** film in 1× PBS (pH 7.4) containing (a) AA, DA, and UA (1 mM AA; 1mM DA; 1 mM UA); (b) PC, AA, DA, and UA (1 mM PC; 1 mM AA; 1mM DA; 1 mM UA); (c) IS, AA, DA, and UA (1 mM IS; 1 mM AA; 1mM DA; 1 mM UA); (d) HA, AA, DA, and UA (1 mM HA; 1 mM AA; 1mM DA; 1 mM UA).


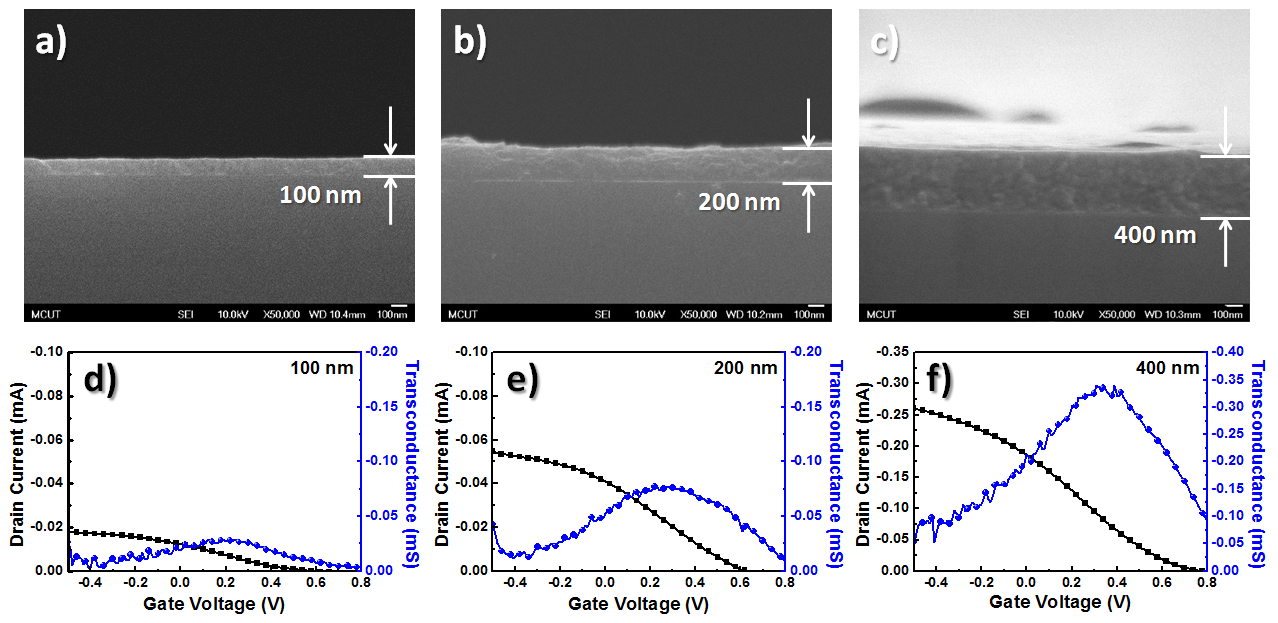


**Figure S4.** Transfer curves and transconductance plotted with respect to *V*_g_ for **P** OECTs with three different thicknesses (100, 200, and 400 nm) of PEDOT:PSS active layer channels [width (W): 1.5 mm; length (L): 5 mm] in 1× PBS (pH 7.4) at a values of *V*_d_ of ‒0.4 V: potential swept from ‒0.5 to +0.8 V.

**Reference:**

[1] Xia, Y., and Ouyang, J. (2012). Significant different conductivities of the two grades of poly (3, 4-ethylenedioxythiophene): poly (styrenesulfonate), Clevios P and Clevios PH1000, arising from different molecular weights. *ACS Appl. Mater. Interfaces* 4, 4131–4140.
